# Selective emergence of antibody-secreting cells in the multiple sclerosis brain

**DOI:** 10.1016/j.ebiom.2023.104465

**Published:** 2023-02-14

**Authors:** Laurens Bogers, Hendrik J. Engelenburg, Malou Janssen, Peter-Paul A. Unger, Marie-José Melief, Annet F. Wierenga-Wolf, Cheng-Chih Hsiao, Matthew R.J. Mason, Jörg Hamann, Jamie van Langelaar, Joost Smolders, Marvin M. van Luijn

**Affiliations:** a Department of Immunology, MS Center ErasMS, Erasmus MC, University Medical Center Rotterdam, 3015 CN, Rotterdam, The Netherlands; b Neuroimmunology Research Group, Netherlands Institute for Neuroscience, 1105 BA, Amsterdam, The Netherlands; c Department of Neurology, MS Center ErasMS, Erasmus MC, University Medical Center Rotterdam, 3015 CN, Rotterdam, The Netherlands; d Department of Viroscience, Erasmus MC, University Medical Center Rotterdam, 3015 CN, Rotterdam, The Netherlands; e Department of Experimental Immunology, Amsterdam Institute for Infection and Immunity, Amsterdam University Medical Centers, 1007 MB, Amsterdam, The Netherlands

**Keywords:** Central nervous system, B-cell maturation, Immunoglobulins, CXCR3, White matter lesions, T cells

## Abstract

**Background:**

Although distinct brain-homing B cells have been identified in multiple sclerosis (MS), it is unknown how these further evolve to contribute to local pathology. We explored B-cell maturation in the central nervous system (CNS) of MS patients and determined their association with immunoglobulin (Ig) production, T-cell presence, and lesion formation.

**Methods:**

*Ex vivo* flow cytometry was performed on post-mortem blood, cerebrospinal fluid (CSF), meninges and white matter from 28 MS and 10 control brain donors to characterize B cells and antibody-secreting cells (ASCs). MS brain tissue sections were analysed with immunostainings and microarrays. IgG index and CSF oligoclonal bands were measured with nephelometry, isoelectric focusing, and immunoblotting. Blood-derived B cells were cocultured under T follicular helper-like conditions to evaluate their ASC-differentiating capacity *in vitro*.

**Findings:**

ASC versus B-cell ratios were increased in post-mortem CNS compartments of MS but not control donors. Local presence of ASCs associated with a mature CD45^low^ phenotype, focal MS lesional activity, lesional Ig gene expression, and CSF IgG levels as well as clonality. *In vitro* B-cell maturation into ASCs did not differ between MS and control donors. Notably, lesional CD4^+^ memory T cells positively correlated with ASC presence, reflected by local interplay with T cells.

**Interpretation:**

These findings provide evidence that local B cells at least in late-stage MS preferentially mature into ASCs, which are largely responsible for intrathecal and local Ig production. This is especially seen in active MS white matter lesions and likely depends on the interaction with CD4^+^ memory T cells.

**Funding:**

Stichting MS Research (19-1057 MS; 20-490f MS), National MS Fonds (OZ2018-003).

**Research in context**

**Evidence before this study**

B cells play a detrimental role in the pathogenesis of multiple sclerosis (MS), which is exemplified by the presence of oligoclonal bands unique to the cerebrospinal fluid (CSF) and the efficacy of anti-CD20 treatment. However, B-cell mechanisms underlying MS pathology are not fully understood yet. Previously, we found that circulating B cells of MS patients are more prone to differentiate into CXCR3-expressing, brain-homing populations. How these B-cell populations further mature and are associated with lesion formation and activity in the CNS is still unknown.

**Added value of this study**

Using single cell suspensions from distinct CNS compartments, the current study reveals that local transition of B cells into antibody-secreting cells (ASCs) is a process that occurs in MS patients and not in controls. Local ASC presence associates with lesion activity, as well as tissue and intrathecal immunoglobulin (Ig) production. Especially, IgG(1,3) gene expression is increased in MS versus control white matter, as well as in the rims of active MS lesions compared to perilesional tissue. From all CNS compartments analysed, ASC versus B-cell ratios only positively correlated with CD4^+^ versus CD8^+^ memory T cell ratios in lesions. The proximity of B cells to CXCR5^+^ T cells in perivascular spaces of MS lesions further suggests that local B cell-to-ASC maturation is locally driven. Taken together, these data provide insights into how B-cell development into ASCs is regulated at different locations within the affected CNS of MS patients.

**Implications of all the available evidence**

Our findings demonstrate that local B-cell maturation into ASCs is a promising target for upcoming drugs that are capable of penetrating the brain. This directly links to our recent findings that CXCR3^+^ B-cell maturation into ASCs can be targeted by the next-generation BTK inhibitor evobrutinib under T_FH_-like *in vitro* conditions. Further studies on the mechanism of action of such compounds will potentially lead to new therapeutic strategies that can be implemented in clinical practice.

## Introduction

B cells induce both humoral and cellular immune responses to protect humans against infections. However, some populations contribute to autoimmune and chronic inflammatory diseases through secretion of autoantibodies and pro-inflammatory cytokines, by recognizing and presenting autoantigens, and/or via formation of ectopic germinal centers.^1^ For more accurate prognosis and effective treatment of such diseases, it is crucial to uncover the triggers, development, and effector functions of disease-related B cells.

Normally, B cells (CD20^+^CD19^+^) develop within the bone marrow and enter the blood circulation as transitional B cells (CD38^high^CD27^−^CD24^high^IgD^+^), which in turn differentiate to naive B cells (CD38^dim/−^CD27^−^IgD^+^). In secondary lymphoid organs, naive B cells are activated to further mature into antigen-specific memory B cells (CD38^dim/−^CD27^+^IgD^low/−^) and eventually develop into antibody-secreting cells (ASC; CD38^high^CD27^high^CD24^−^IgD^−^), comprising plasmablasts and plasma cells that actively produce high-affinity immunoglobulins (Ig).^2^^,^^3^ ASCs usually lose CD20 expression and show variable levels of CD19.^3^ In autoimmune diseases such as systemic lupus erythematosus, central B-cell tolerance checkpoints are defective, allowing potentially pathogenic B cells to enter the circulation.^4^ In particular, B cells expressing the transcription factor T-bet and its surrogate marker CXCR3 have been implicated in systemic autoimmunity.^5^, ^6^, ^7^, ^8^ Their development is likely driven by both T cell-dependent (e.g. CD40L, IL-21 and IFN-γ) and -independent (e.g. TLR7/9 ligands) mechanisms, which define further maturation into memory and ASC populations. How T-bet^+^ B cells are involved in organ-specific chronic inflammatory diseases is less well understood, especially in humans.

B cells have a central role in the pathophysiology of multiple sclerosis (MS), a chronic inflammatory disease of the central nervous system (CNS).^9^, ^10^, ^11^ Depletion of circulating B cells with anti-CD20 therapies appears to be efficacious in MS, which emphasizes the relevance of these cells for the disease process.^12^^,^^13^ At diagnosis, about 90% of MS patients show oligoclonal bands (OCB) unique to the cerebrospinal fluid (CSF), indicating intrathecal Ig production by multiple B-cell clones.^14^^,^^15^ In fact, B-cell aggregates have been found within meninges and parenchyma of the CNS and associate with adverse clinical outcomes in MS.^16^, ^17^, ^18^ B-cell aggregates are present in 30–40% of progressive MS patients.^9^

In contrast to most autoimmune diseases, only peripheral and not central B-cell tolerance is defective in MS patients.^19^ Naive B cells that have escaped this control most likely mature and differentiate into T-bet^+^ class-switched memory B cells by interacting with infectious triggers and IFN-γ-producing CD4^+^ T follicular helper (T_FH_) cells.^20^, ^21^, ^22^, ^23^ Within the periphery, these B cells mainly function as potent antigen presenting cells to stimulate IFN-γ-producing brain-homing CD4^+^ T cells in MS.^24^ In addition, T-bet^+^ class-switched memory B cells express high levels of CXCR3, enabling them to enter the CNS.^20^^,^^23^^,^^25^ B-cell populations are likely reactivated in so-called meningeal tertiary lymphoid structures and white matter perivascular spaces.^17^^,^^20^^,^^26^^,^^27^

Currently, it is unclear how CXCR3^+^ B cells further evolve and behave subsequent to their migration into the CNS and whether this process corresponds to local MS pathology. Here, we explored the transition of *ex vivo* B to antibody-secreting cells in distinct CNS compartments of MS and control brain donors, which was associated with lesion activity, local Ig production and the presence of CD4^+^ T cells.

## Methods

### Patients

For *ex vivo* phenotyping of B cells, we freshly obtained post-mortem blood, CSF, meninges and white matter tissue from MS and non-demented control (NDC) brain donors (n = 28 and n = 10, respectively) (Netherlands Brain Bank, Amsterdam, The Netherlands). For gene expression analyses, post-mortem MS normal appearing white matter (NAWM; n = 17) and white matter lesions (n = 15) as well as NDC white matter (n = 14) were used from additional cohorts.^28^^,^^29^ For immunofluorescence staining, post-mortem MS white matter lesions (n = 6) were used. For *ex vivo* phenotyping of T cells, post-mortem MS white matter and white matter lesions (n = 4) were freshly obtained. Causes of death among MS patients involved a wide variety of events, including legal euthanasia, pneumonia, MS, sepsis, cardiac arrest, and others. In addition, blood samples were obtained from a large cohort of untreated progressive MS patients (n = 7) and healthy controls (n = 7) at MS center ErasMS (Erasmus MC, Rotterdam, The Netherlands). The sex of MS donors included in this study reflects the female bias among the patient population. Clinical information on all subjects was extracted from patient records of the MS center ErasMS and the Netherlands Brain Bank and is summarized in Table 1. Individual information of the included brain donors can be found in Supplementary Tables S1–S5. Primary material was obtained in the period between 1996 and 2021.

**Table 1:** Summarized clinical information of MS and control brain and blood donors included in this study.

Cohorts	Subject, n	Female sex, n (%)	Age in years, median (IQR)^a^	PMD in hours, median (IQR)^b^	pH-value of CSF, median (IQR)
*Ex vivo* B cells, CNS and blood					
MS	28	22 (79%)	65 (52.5–67.5)	8.4 (6.9–9.1)	6.55 (6.40–6.64)
1. Peripheral blood	22				
2. CSF	20				
3. Meninges	23				
4. White matter	19				
5. Lesion	13				
NDC	10	7 (70%)	83 (72–86)	7.6 (6.3–8.8)	6.61 (6.50–6.89)
1. Peripheral blood	7				
2. CSF	5				
3. Meninges	10				
4. White matter	7				
OCB and IgG index, CSF and blood					
MS	24	19 (79%)	65 (52.5–67.5)	8.4 (6.6–9.1)	6.55 (6.40–6.67)
Gene expression, brain tissue (WM)					
MS	17	17 (100%)	64 (48–70.5)	7.8 (6.7–9.5)	6.40 (6.26–6.55)
NDC	14	11 (79%)	60.5 (54–68)	7.3 (6.5–9.3)	6.90 (6.57–7.12)
Gene expression, brain tissue (lesions)					
MS (I)	8	6 (75%)	65 (53.5–73.5)	9.1 (7.9–10.1)	6.44 (6.26–6.50)
MS (CA)	7	5 (71%)	48 (43–53)	8.2 (7.0–10.9)	6.48 (6.23–6.55)
Immunofluorescence, brain tissue (lesions)					
MS	6	3 (50%)	53 (53–56)	8.5 (6.6–10.0)	6.41 (6.29–6.81)
*Ex vivo* T cells, brain tissue (WM, lesions)					
MS	4	2 (50%)	66 (56–72)	6.5 (5.6–7.35)	6.53 (6.45–6.68)
*In vitro* stimulated B cells, blood					
MS	7	4 (57%)	56 (47–63)	NA	NA
HC	7	4 (57%)	43 (23–54)	NA	NA

IQR = interquartile range; PMD = post-mortem delay; CNS = central nervous system; CSF = cerebrospinal fluid; WM = white matter; OCB = oligoclonal bands; MS = multiple sclerosis; NDC = non-demented control; HC = healthy control; I = inactive MS; CA = chronically active MS; NA = not applicable.

a At point of death or sampling.

b Time between death and autopsy.

### Isolation of mononuclear cells from peripheral blood

As described previously,^23^ peripheral blood from MS patients and healthy controls was collected via venipuncture into vacutainer CPT tubes, while peripheral blood from MS and NDC brain donors was obtained via cardiac puncture. Peripheral blood mononuclear cells (PBMC) were isolated according to manufacturer's protocol and either freshly used for multicolour flow cytometry or frozen in liquid nitrogen for later use. Plasma samples were stored at −80 °C for later use.

### Isolation of mononuclear cells from CNS compartments

CSF, leptomeninges and brain tissues from MS and NDC brain donors were processed and mononuclear cells were isolated as reported previously.^23^^,^^30^ CSF supernatants were stored at −80 °C for later use. Brain tissues were distinguished macroscopically and with magnetic resonance imaging (MRI) into white matter and white matter lesions at the Netherlands Brain Bank. Macroscopically representative brain tissues were snap-frozen and stored at −80 °C for *in situ* lesion staging. For digestion, tissues were treated with 75 μg/mL liberase and 100 U/mL DNAse I (Roche, Basel, Switzerland). Mononuclear cells from all CNS compartments were diluted in phosphate-buffered saline (Lonza, Verviers, Belgium) with 0.1% bovine serum albumin and further used for multicolour flow cytometry.

Brain tissues from MS donors used for *ex vivo* T cell phenotyping were processed and mononuclear cells were isolated as described before.^31^ For digestion, brain tissues were first mechanically dissociated and then treated with 300 U/mL collagenase IV (Worthington, Lakewood, United States) and 10 U/mL DNAse I (Roche). Mononuclear cells were used for multicolour flow cytometry.

### In vitro stimulation of human B cells

Thawed PBMCs (5 × 10^4^ cells per well) from progressive MS patients and healthy controls were cocultured *in vitro* with murine NIH3T3 fibroblasts highly expressing human CD40 ligand (3T3-CD40L cells; 15 × 10^3^ cells per well) and soluble IL-21 (50 ng/mL; Thermo Fisher Scientific, Landsmeer, The Netherlands) in B-cell medium for 6 and 11 days as described before.^23^ Afterwards, the cells were used for multicolour flow cytometry. Supernatants from day 6 were stored at −80 °C.

### Multicolour flow cytometry

Thirteen colour-based flow cytometry was performed using a variety of fluorochrome-labelled monoclonal anti-human antibodies (Supplementary Table S6) as described previously.^23^ Fixation and permeabilization was done using the Foxp3 fixation/permeabilization kit according to manufacturer's protocol (eBioscience, San Diego, United States).

### In situ lesion staging

Macroscopically defined white matter and white matter lesions from MS brain donors were characterized *in situ* using a Klüver-Barrera staining and subsequent HLA-DP/DQ/DR immunohistochemistry. As previously described, different white matter lesion stages can be distinguished microscopically (Supplementary Table S7).^32^, ^33^, ^34^ First, 6–7 μm cryosections were fixed in dehydrated acetone. We followed the protocol as previously described for Klüver-Barrera staining^35^ and HLA staining.^34^ Images were captured at 20× magnification, using an AxioScanZ1 (Zeiss, Oberkochen, Germany).

### Immunofluorescence and confocal imaging

Formalin-fixed paraffin embedded sections (8 μm) of MS white matter lesions were stained for CD19, CD3 and CXCR5. Antigen retrieval was performed through microwave treatment with citraconic anhydride (Ph 7.6). Sections were incubated overnight with primary antibody (CD19 (rabbit), 1:200, 11501, Biolegend; CD3 (rat), 1:50, ab11089, Abcam; CXCR5 (mouse), 1:100, MAB190, RD Systems). Endogenous peroxidase activity was quenched in a 1% H_2_O_2_ solution. Then, slides were incubated with rat absorbed biotinylated anti-mouse IgG (1:400; BA-2001, Vector Laboratories, Burlingame, United States), followed by incubation with Vectastain Elite ABC kit (1:800, PK-6100, Vector Laboratories) and biotinylated tyramide (1:10000; SML2135, Sigma–Aldrich, Saint Louis, United States) with 0.001% H_2_O_2_. Then, sections were incubated with the fluorophores anti-rat Cy3 (1:400; 712-166-153, Jackson ImmunoResearch, Uden, The Netherlands), Streptavidin Alexa Fluor 647 (1:600; 016-600-084, Jackson) and anti-rabbit Alexa Fluor 488 (1:400; 711-546-152, Jackson). Last, sections were incubated with 0.1% Sudan Black B and subsequently with Hoechst 33342 (1:1000; H3570, Invitrogen, Paisley, United Kingdom). Imaging was performed using a Leica microscope TSA SP8 X and Leica Applications Suite X software at 63× magnification. Quantification of images was conducted through manual counting of CXCR5-expressing CD3^+^ cells in relation to T- and B-cell contact using QuPath (version 0.3.2).

### RNA isolation, lesion dissection and microarray pre-processing

As described by Melief et al. and Hendrickx et al.,^28^^,^^29^ 20 μm cryosections of MS and NDC brain tissues were stained with proteolipid protein (PLP) (Serotec, Oxford, United Kingdom) and HLA-DP/DQ/DR (DAKO, Glostrup, Denmark) to identify NAWM and distinguish chronic active and inactive MS lesions. Laser dissection microscopy was used to separate the rim of MS lesions and perilesional tissue. From these tissues, RNA was isolated, labelled and hybridized. Finally, microarray scans were created using an Agilent DNA Microarray Scanner and quantified with Agilent Feature Extraction software (version 9.5.3.1.).^28^^,^^29^

### Enzyme-linked immunosorbent assay (ELISA)

As described previously,^36^ IgM, IgA and IgG levels were determined in supernatants from *in vitro* B-cell cocultures using an ELISA. Plates were coated with goat anti-human IgM, IgG or IgA (1 mg/mL; Southern Biotech, Uithoorn, The Netherlands). Horseradish peroxidase conjugated rabbit anti-human IgM (Jackson), goat anti-human IgG (Thermo Fisher Scientific) or goat anti-human IgA (Jackson) were used to detect bound antibodies.

### OCB and IgG index measurement in plasma and CSF

Paired plasma and CSF samples from MS brain donors, stored at −80 °C, were used for assessing the presence of OCBs and measuring the IgG index. IgG index was determined for all MS brain donors by applying nephelometry and OCBs were analysed in all samples using isoelectric focusing and IgG immunoblotting.^37^

### Statistical analysis

GraphPad Prism 8.0.1 (GraphPad Software, San Diego, United States) was used for interpreting all datasets and performing statistical analyses. For two group data, paired or unpaired t-tests were performed if assumptions were met. Otherwise, equivalent non-parametric tests were performed (Wilcoxon signed rank & Mann Whitney U tests). For all data with more than two groups, assumptions were violated and non-parametric Kruskal–Wallis (unpaired) and Friedman (paired) tests were performed. Post-hoc pairwise comparisons were performed using the Dunn's post-hoc test. In addition, all correlations were assessed by Spearman correlation coefficient. p-values of <0.05 were considered significant (two-tailed). Statistical significance is represented within each graph. All unpaired data are represented as mean ± standard error of the mean (SEM).

### Ethics

All patients provided written informed consent. Study protocols were approved by the medical ethics committee of the Erasmus Medical Center (2021-0251; 2021-0946) and the VU University Medical Center (2009-148).

### Role of funders

Funding by Stichting MS Research (19-1057 MS; 20-490f MS) and National MS Fonds (OZ2018-003) made it possible to conduct the study. However, the funders were not involved in the study design, data collection, analysis and interpretation, or writing the paper.

## Results

### Enhanced B cell-to-ASC transition in distinct CNS compartments of MS and not control donors

**Fig. 1:**
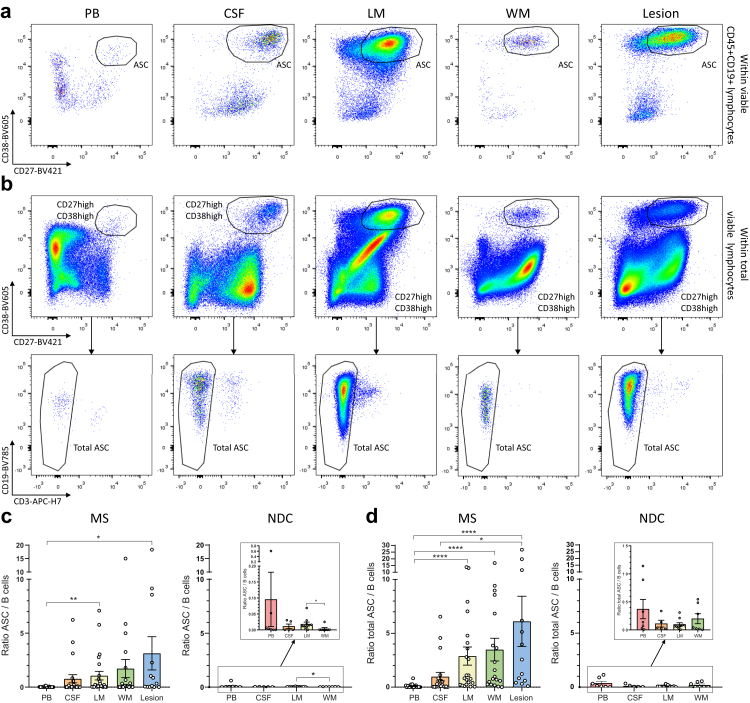
***Ex vivo* ASCs are enriched in relation to B cells in the CNS of MS patients.** (**a**) Representative flow cytometry dot plots with gating of *ex vivo* CD38^high^CD27^high^ ASCs within viable CD45^+^CD19^+^CD3^−^ lymphocytes from freshly obtained paired post-mortem blood, CSF, leptomeninges, white matter and white matter lesions of MS brain donors. (**b**) Representative flow cytometry dot plots with gating of *ex vivo* CD38^high^CD27^high^CD3^−^ cells (total ASCs) within total viable lymphocytes from freshly isolated post-mortem peripheral and CNS compartments of MS brain donors. (**c**, **d**) Quantifications of ASC/B-cell ratios and total ASC/B-cell ratios in peripheral and CNS compartments of MS brain donors (n = 28) and NDCs (n = 10). Data are presented as the mean ± standard error of the mean (SEM). Data were analysed using Kruskal–Wallis and Dunn's post-hoc tests. ∗p < 0.05; ∗∗p < 0.01; ∗∗∗p < 0.001; ∗∗∗∗p < 0.0001. ASC = antibody-secreting cell; CSF = cerebrospinal fluid; LM = leptomeninges; MS = multiple sclerosis; NDC = non-demented control; PB = peripheral blood; WM = white matter.

To address whether B-cell maturation is different in the MS brain, we compared the ratios of *ex vivo* ASCs (CD45^+^CD19^+^CD3^−^CD38^high^CD27^high^) and B cells (CD45^+^CD19^+^CD3^−^CD38^dim/−^) within the viable lymphocyte gate between single cell suspensions from post-mortem blood, CSF, meninges and white matter compartments of 28 late-stage MS donors and 10 non-demented control (NDC) donors using multicolour flow cytometry (Fig. 1a). Since human tissue-plasma cells can lose CD45 and/or CD19 expression upon maturation,^38^ we also gated cells that displayed an ASC phenotype (CD38^high^CD27^high^CD3^−^) within viable lymphocyte fractions irrespective of the presence of CD45 and CD19 (total ASCs; Fig. 1b). Despite the fact that liberase as part of our single-cell isolation protocol for brain tissues reduced CD27 expression by B cells (Supplementary Fig. S1a–c), we were able to detect high numbers of CD38^high^CD27^high^ ASCs, especially in meninges and white matter lesions from MS donors (Fig. 1a and b).

Although ASC counts were clearly the highest in meninges (Supplementary Fig. S2a and b), the ASC/B-cell ratio was increased in all CNS compartments compared to the blood from MS donors, with the highest proportion within macroscopically defined lesions (Fig. 1c). These differences were even more pronounced for the total ASC/B-cell ratio, suggesting that ASCs lacking CD45 and CD19 expression contribute to the increase of ASCs within CNS compartments (Fig. 1d). In NDCs, both CD19^+^ ASC and total ASC/B-cell ratios did not differ between blood and CNS compartments (Fig. 1c and d), suggesting a local shift in B-cell maturation towards ASCs in MS but not control donors.

### Local ASC presence corresponds with lesion activity and Ig expression in MS brain tissue

**Fig. 2:**
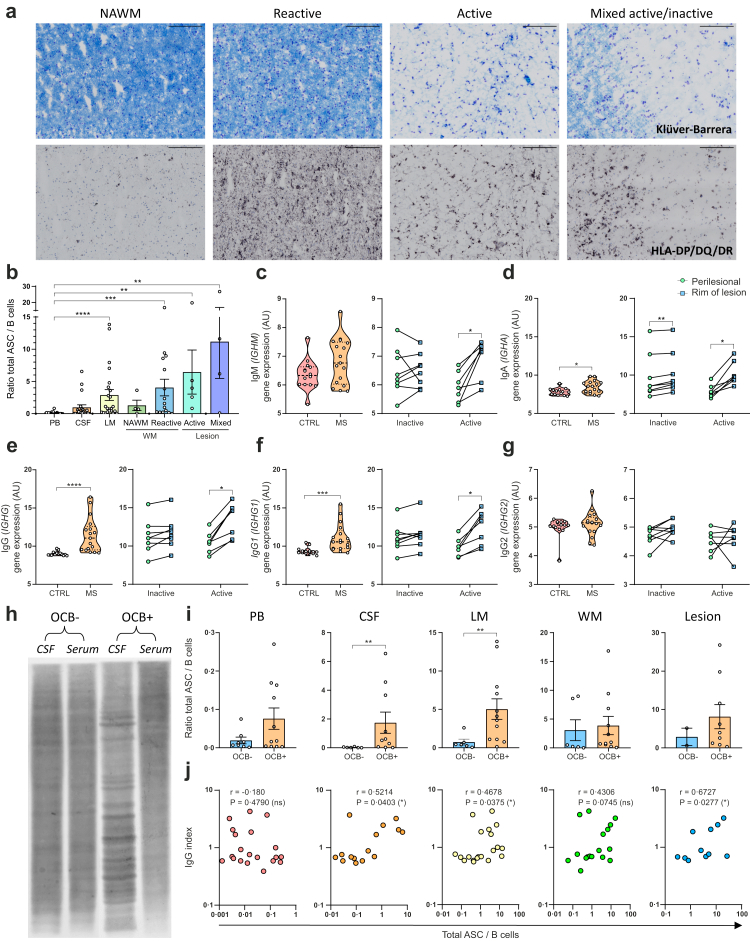
**ASCs in the MS CNS associate with lesion activity, local Ig expression and intrathecal IgG production.** (**a**) Representative pictures (20× magnification) of Klüver-Barrera staining and HLA-DP/DQ/DR immunohistochemistry on macroscopically defined white matter and white matter lesions from MS brain donors. Scale bars are 200 micron. White matter is distinguished into NAWM and reactive sites, while white matter lesions are divided into active and mixed active/inactive lesions. (**b**) Quantifications of total ASC/B-cell ratios in these different brain tissues compared to peripheral blood, CSF and leptomeninges. (**c**–**g**) Normalized gene expression levels of *IGHM*, *IGHA*, *IGHG*, *IGHG1* and *IGHG2* in post-mortem control white matter (n = 14) and MS NAWM (n = 17) as well as in post-mortem perilesional tissue and rims of inactive (n = 8) and active (n = 7) MS white matter lesions, measured with a microarray. (**h**) Representative pictures of immunoblotting with post-mortem CSF and serum from MS brain donors with and without OCBs. (**i**) Quantification of total ASC/B-cell ratios in post-mortem peripheral blood, CSF, leptomeninges, white matter and white matter lesions from OCB-negative and -positive MS patients. (**j**) Correlation plots showing the link between IgG index and total ASC/B-cell ratios in post-mortem peripheral and CNS compartments from MS brain donors. Data are presented as the mean ± standard error of the mean (SEM). p-values were calculated using Kruskal–Wallis and Dunn's post-hoc tests (**b**), Mann–Whitney U tests (**c**–**g**, **i**) and Wilcoxon signed-rank tests (**c**–**g**). Correlation plots were analysed by calculating Spearman correlation coefficients (**j**). ∗p < 0.05; ∗∗p < 0.01; ∗∗∗p < 0.001; ∗∗∗∗p < 0.0001. ASC = antibody-secreting cell; AU = arbitrary units; CSF = cerebrospinal fluid; CTRL = control; LM = leptomeninges; MS = multiple sclerosis; NAWM = normal appearing white matter; OCB = oligoclonal band; PB = peripheral blood; WM = white matter.

To further study the association of this shift with lesion activity, we classified our MS white matter tissues based on immunohistochemical staining of both myelin (Klüver-Barrera) and human leukocyte antigen DP, DQ and DR (HLA-DP/DQ/DR; Fig. 2a).^39^^,^^40^ No inactive lesions appeared present in our tissue selection. Normal appearing white matter (NAWM; n = 4), reactive sites (n = 15), active lesions (n = 5) and mixed active/inactive lesions (n = 4) were identified (Supplementary Table S7). Total ASC/B-cell ratios were found to be significantly higher in reactive sites, active lesions and mixed active/inactive lesions compared to peripheral blood. Despite the low sample size, these ratios seemed to be substantially lower in NAWM (Fig. 2b). To examine whether presence of ASCs in active lesions also corresponds to Ig production in lesions, we next analysed whole-tissue microarray data from MS NAWM and control white matter, as well as dissected perilesional tissues and rims of inactive and active MS white matter lesions, for gene expression of IgM (*IGHM*), IgA (*IGHA*), and IgG (*IGHG*, *IGHG1*, *IGHG2* and *IGHG3*).^28^^,^^29^ Total IgM gene expression was slightly higher in MS NAWM compared to white matter from control donors, while total IgA and IgG gene expression was significantly increased (Fig. 2c–e). IgG/IgA and IgG/IgM gene expression ratios were also elevated in MS NAWM compared to control white matter, suggesting a more pronounced increase in IgG relative to IgA and IgM (Supplementary Fig. S3a–c). Besides, total IgM, IgA and IgG gene expression was significantly higher in the rim compared to perilesional tissue of active MS lesions, while this was not seen for inactive MS lesions (Fig. 2c–e). Interestingly, the elevated IgG gene expression was mainly due to IgG1 (*IGHG1*; Fig. 2f) and IgG3 (*IGHG3*; Supplementary Fig. S3d), but not IgG2 (*IGHG2*; Fig. 2g), which is in line with the presumed IgG1- and IgG3-switched phenotype of T-bet^+^ B cells both in MS and during viral infections.^23^^,^^41^ These findings corroborate our flow cytometry data, showing not only cells with an ASC phenotype but also corresponding Ig expression to be enriched in active MS white matter lesions.

### B cell-to-ASC transition in the MS CNS is associated with increased intrathecal IgG production

To further link the *ex vivo* presence and spatial distribution of ASCs to IgG production within the CNS of MS patients, we analysed IgG index and OCBs in CSF of included MS brain donors when available (n = 24; Fig. 2h). In CSF and meninges, total ASC/B-cell ratios were significantly higher in OCB^+^ than in OCB^−^ patients (Fig. 2i). Moreover, total ASC/B-cell ratios in all CNS compartments, but not in blood, positively correlated with IgG index in MS patients (Fig. 2j). Similar correlations with OCBs and IgG index were observed when analysing ASC counts (Supplementary Fig. S2c and d). These data implicate that ASC enrichment in MS CNS contributes to enhanced intrathecal oligoclonal IgG production.

To further consolidate their role as high producers of Igs, we analysed CD38^high^CD27^high^ ASCs for intracellular IgM, IgG and IgA expression both *ex vivo* and *in vitro* using multicolour flow cytometry (Supplementary Fig. S4a). In both the *ex vivo* and *in vitro* data, we confirmed that CD38^high^CD27^high^ ASCs are significantly more IgG^+^ and are higher IgG, IgM, and IgA producers compared to CD19^+^CD38^dim/−^ B cells (Supplementary Fig. S4b–e). The percentage of CD138^+^ cells was significantly higher in CD38^high^CD27^high^ ASCs compared to CD19^+^CD38^dim/−^ B cells, supporting their identity as ASCs (Supplementary Fig. S4f and g). Additionally, CD138 (*SDC1*) gene expression was assessed in brain tissue with a microarray. Gene expression did not differ between MS and control white matter and no differences were seen between the rim and perilesional tissue of active MS lesions, indicating that the earlier described increase of Ig gene expression in these tissues is independent of CD138 expression (Supplementary Fig. S4h).

### MS brain ASCs show a mature CD45^low^ phenotype, while MS CSF seems to contain a more active subset

**Fig. 3:**
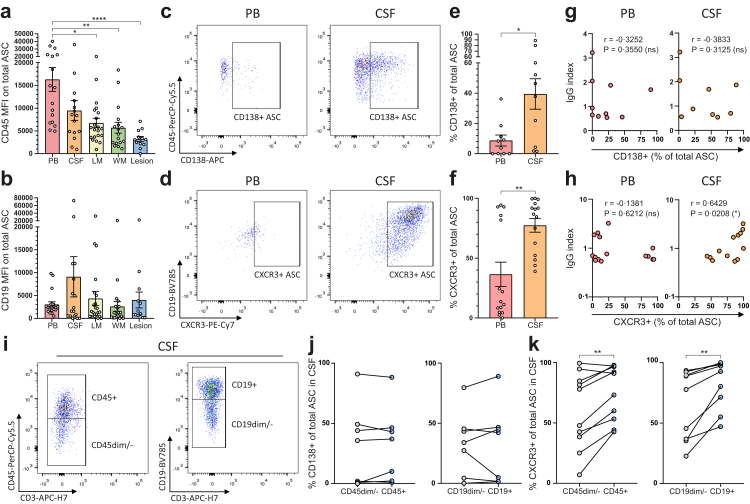
**ASCs in MS CNS tissues show a mature phenotype, while CSF contains a more active subset of ASCs.** (**a**, **b**) Quantifications of CD45 and CD19 MFI on ASCs from post-mortem peripheral blood and CNS compartments of MS brain donors. (**c**, **d**) Representative flow cytometry dot plots with gating of CD138^+^ and CXCR3^+^ cells within total ASCs from peripheral blood and CSF. (**e**, **f**) CD138^+^ and CXCR3^+^ percentages within total ASCs from peripheral blood and CSF. (**g**, **h**) Correlation plots showing the link between IgG index and CXCR3 expression on total ASCs in post-mortem peripheral blood and CSF. (**i**) Representative flow cytometry dot plots with gating of CD45 and CD19 negative and positive populations in total ASCs from post-mortem CSF. (**j**, **k**) Paired CD138^+^ and CXCR3^+^ percentages within these CD45 and CD19 negative and positive ASC populations. Data are presented as the mean ± standard error of the mean (SEM). Data were analysed by performing Kruskal–Wallis and Dunn's post-hoc tests (**a**, **b**), Mann–Whitney U tests (**e**, **f**) and paired t-tests (**j**, **k**). Correlation plots were analysed by calculating Spearman correlation coefficients. ∗p < 0.05; ∗∗p < 0.01; ∗∗∗p < 0.001; ∗∗∗∗p < 0.0001. ASC = antibody-secreting cell; CSF = cerebrospinal fluid; LM = leptomeninges; MFI = median fluorescence intensity; MS = multiple sclerosis; PB = peripheral blood; WM = white matter.

Since ASC maturation may determine local Ig production, we evaluated surface levels of CD45 and CD19 on ASCs in CNS compartments of MS brain donors. ASCs can lose expression of CD45 and CD19 during maturation.^38^ Interestingly, the median fluorescence intensity (MFI) of CD45 gradually decreased on ASCs closer localized to white matter and was significantly lower in white matter and MS white matter lesions compared to peripheral blood (p = 0.0018 and p ≤ 0.0001, respectively), indicating a highly mature ASC phenotype in the MS brain (Fig. 3a). CD19 was more variably expressed, but was lower on ASCs in meninges and brain tissue compared to CSF (Fig. 3b). Reduced expression of CD45 and CD19 were not due to the isolation protocol, as *ex vivo* B cells from healthy donor blood did not show reduced marker expression after treatment with liberase (Supplementary Fig. S1d and e). Unfortunately, liberase reduced expression of CD138 and CXCR3, excluding analysis of these markers in CNS tissues (Supplementary Fig. S1f and g).

To further associate the phenotype of ASCs in the MS CSF to Ig production, expression of CD138 and CXCR3 was compared between CSF and blood of MS brain donors (Fig. 3c and d). Together with CD19, which was highest on CSF ASCs, CD138 and CXCR3 are likely indicative for Ig producing capacity.^38^^,^^42^ While CD138^−^ subsets predominated both compartments, the percentage of CD138^+^ ASCs was significantly higher in CSF compared to blood (p = 0.0137; Fig. 3e). This was similar for CXCR3 expression (p = 0.0048), consistent with the CXCR3^+^ phenotype of CNS-infiltrating CD19^+^ cells in MS (Fig. 3f).^23^ In contrast to CD138 (Fig. 3g), CXCR3 levels on CSF ASCs positively correlated with IgG index (r = 0.6429; p = 0.0208; Fig. 3h), suggesting that intrathecal IgG production by ASCs may depend on CXCR3 expression in line with our previous observations.^42^ In addition, CD45 and CD19 positive and negative ASC populations were identified in post-mortem CSF of included MS brain donors to investigate whether CD138 and CXCR3 are differently expressed on intrathecal ASCs with a mature and immature phenotype (Fig. 3i). Interestingly, CD138 expression was not different between these groups, while CXCR3 expression was higher on CD45^+^ and CD19^+^ ASCs (Fig. 3j and k). Taken together, CXCR3 expression is likely highest on ASCs showing a less mature phenotype and a strong Ig producing capacity.

### *In vitro* maturation of peripheral B cells to ASCs is not enhanced for MS patients

**Fig. 4:**
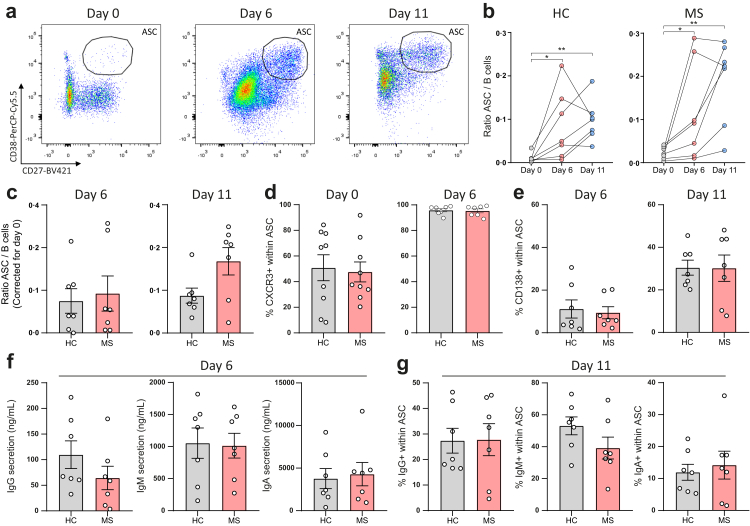
**No difference in peripheral B cell-to-ASC differentiation between healthy controls and MS patients under T**_**FH**_**-like conditions *in vitro*.** PBMCs from healthy controls (n = 7) and progressive MS patients (n = 7) were cocultured with CD40L-attached 3T3 fibroblasts and stimulated with soluble IL-21 (T_FH_-like conditions) for 6 and 11 days, followed by multicolour flow cytometry. (**a**) Representative flow cytometry dot plots with gating of CD38^high^CD27^high^ ASCs within viable CD19^+^CD3^-^ lymphocytes on day 0, 6 and 11. (**b**) Paired ASC/B-cell ratios on day 0, 6 and 11 for both healthy controls and MS patients. (**c**) Quantifications of ASC/B-cell ratios on day 6 and 11 for both groups, corrected for values on day 0. (**d**) Percentages of CXCR3^+^ ASCs on day 0 and 6 for healthy controls and MS patients. (**e**) Percentages of CD138^+^ ASCs on day 6 and 11 for both groups. (**f**) Quantifications of IgG, IgM and IgA secretion in supernatants on day 6, measured with an ELISA. (**g**) Percentages of intracellular producing IgG^+^, IgM^+^ and IgA^+^ ASCs on day 11. Data are presented as the mean ± standard error of the mean (SEM). Data were analysed with Friedman and Dunn's post hoc tests (**b**) and Mann–Whitney U tests (**c–g**). ∗p < 0.05; ∗∗p < 0.01; ∗∗∗p < 0.001; ∗∗∗∗p < 0.0001. ASC = antibody-secreting cell; HC = healthy control; MS = multiple sclerosis.

In an attempt to explore the drivers of B cell-to-ASC transition in the MS brain, we first examined whether peripheral B cells from MS patients are intrinsically more capable of differentiating into ASCs. PBMCs from MS patients and healthy controls were cocultured *in vitro* under T_FH_-like conditions to mimic B- and T-cell interaction, followed by multicolour flow cytometry on day 6 and 11 (Fig. 4a).^23^ In both MS patients and healthy controls, increased ASC outgrowth was seen on day 6 and 11 (Fig. 4b). Differentiation into ASCs did not significantly differ between MS patients and healthy controls, although a trend was observed on day 11, leaning towards higher ASC outgrowth from B cells of MS patients (Fig. 4c). This trend could not be explained by patient characteristics nor by differences in the presence of ASCs *ex vivo*. Furthermore, the obtained ASC phenotype as measured by CD138 and CXCR3 expression did not differ between both groups (Fig. 4d and e). Moreover, IgG, IgM and IgA secretion profiles and intracellular production were not significantly different at day 6 and 11, respectively (Fig. 4f and g). These data demonstrate that at least in the circulation, the capacity of B cells to mature into ASCs is not different in MS.

### Higher age correlates with more circulating but less CNS-residing ASCs in MS

**Fig. 5:**
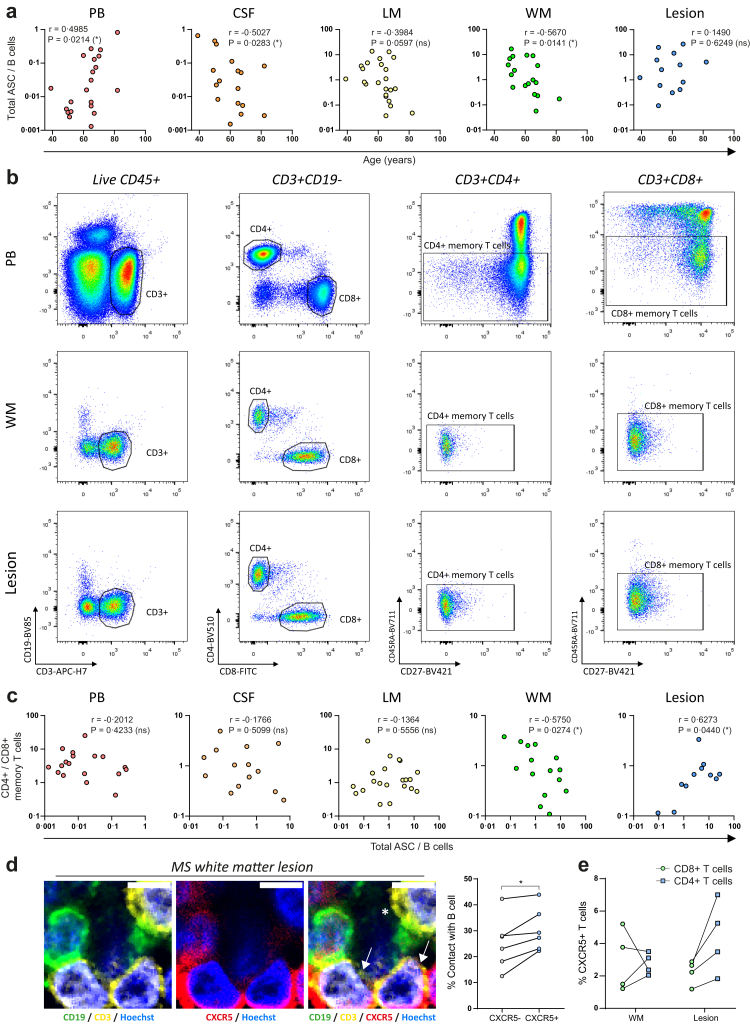
**ASCs in MS lesions associate with local presence of CD4**^**+**^**memory T cells.** (**a**) Correlation plots showing the link between total ASC/B-cell ratios and age in peripheral blood, CSF, leptomeninges, white matter and white matter lesions of MS brain donors. (**b**) Representative flow cytometry dot plots with the gating strategy for identifying CD4^+^CD45RA^−^ and CD8^+^CD45RA^−^ memory T cells (CD3^+^CD19^−^) in viable CD45^+^ lymphocytes from peripheral blood, white matter and white matter lesions of MS brain donors. (**c**) Correlation plots showing the link between CD4^+^/CD8^+^ memory T cell ratios and total ASC/B-cell ratios in peripheral and CNS compartments. (**d**) Confocal images (63× magnification) of CD19 (green), CD3 (yellow) and CXCR5 (red) immunofluorescence staining on MS white matter lesions showing local interplay between CD19^+^ B cells and CD3^+^CXCR5^+^ T cells. Hoechst (blue) was used for staining nuclei. The white arrows indicate CD3^+^CXCR5^+^ T cells. The asterisk indicates a CD3^+^CXCR5^−^ T cell. Scale bars are 5 micron. B-cell contact within total CXCR5^+^ and CXCR5^−^ T cells is quantified in proximity of cuffs within MS white matter lesions (n = 6). (**e**) CXCR5^+^ percentages are determined within total CD4^+^ and CD8^+^ T cells from MS white matter and white matter lesions (n = 4) using flow cytometry. Correlation plots were analysed by calculating Spearman correlation coefficients (**a**, **c**). p-values were calculated using paired t-tests (**d**) and Wilcoxon signed-rank tests (**e**). ∗p < 0.05; ∗∗p < 0.01; ∗∗∗p < 0.001; ∗∗∗∗p < 0.0001. ASC = antibody-secreting cell; CSF = cerebrospinal fluid; LM = leptomeninges; MS = multiple sclerosis; PB = peripheral blood; WM = white matter.

Next, we addressed B cell-extrinsic factors as potential drivers for B-cell maturation into ASCs in the MS brain. CXCR3(T-bet)^+^ B cells, which are prone to differentiate into ASCs,^36^^,^^42^ show a phenotype that has previously been linked to accumulation in murine and human peripheral blood with age.^6^^,^^43^, ^44^, ^45^ We observed a positive correlation between age and total ASC/B-cell ratios in peripheral blood (r = 0.4985; p = 0.0214), while a negative correlation was found in CSF (r = −0.5027; p = 0.0283), meninges (r = −0.3984; p = 0.0597) and white matter (r = −0.5670; p = 0.0141). However, this correlation was not seen in white matter lesions (r = 0.1490; p = 0.6249), suggesting that ASC presence in MS lesions is independent of age (Fig. 5a).

### The presence of CD4^+^ T cells is associated with an increased B cell-to-ASC transition in MS lesions

Local T-cell presence may also be a determining factor for ASCs in the CNS of MS patients, with interaction of B and T cells in a follicle-like environment.^20^ Therefore, *ex vivo* CD4^+^ and CD8^+^ memory T cells were identified within post-mortem peripheral blood and CNS compartments of MS brain donors using multicolour flow cytometry (Fig. 5b). In white matter, a relatively lower ASC/B-cell ratio correlated with an increased fraction of CD4^+^ relative to CD8^+^ memory T cells (r = −0.5750; p = 0.0274). Conversely, in MS white matter lesions, relatively more ASCs were found in patients with a higher abundance of CD4^+^ memory T cells (r = 0.6273; p = 0.0440) (Fig. 5c). These findings suggest that B-cell maturation into ASCs depends on the local presence of CD4^+^ memory T cells with differential regulation in the context of MS white matter and white matter lesions. CXCR5 is a chemokine receptor that is important for B- and T-cell homing into follicles and characterizes a subset of T cells which can provide B cell-maturing stimuli.^46^ We explored the local interaction between B cells and follicular-like T cells in MS white matter lesions with immunofluorescence staining. CD3^+^CXCR5^+^ T cells were observed in direct contact with CD19^+^ B cells and CXCR5^+^ T cells were found to be more frequently in contact with B cells compared to CXCR5^−^ T cells (Fig. 5d). Accordingly, we observed higher expression of CXCR5 on CD4^+^ compared to CD8^+^ T cells in MS white matter lesions, but not in white matter, suggesting local CD4^+^ T cells to have T_FH_-like characteristics (Fig. 5e).

## Discussion

In this study, we demonstrated local maturation to and persistence of ASCs as a hallmark of the MS brain, most abundantly in white matter lesions. B-cell maturation as analysed in CNS compartments of people with MS was associated with *in situ* enhanced lesion activity and both intrathecal as well as local *in situ* IgG production. Not only the increased presence of ASCs, but also their expression of CXCR3 at different locations of the CNS was revealed as a potential determinant for intrathecal IgG production. B-cell maturation in MS lesions seemed dependent on local interaction with CD4^+^ memory T cells. Overall, we provide a complete overview of the ASC and B-cell phenotype and distribution in distinct compartments of the affected (MS) and unaffected (control) human brain. These findings provide a direct link between MS B-cell phenotype, antibody-secreting function, and local interplay with T cells, which adds to existing indirect evidence in providing a rationale for therapies targeting these mechanisms.

CNS-infiltrating CXCR3^+^ B cells have been implicated in MS and further development of this B-cell population into ASCs is thought to be important for local pathology.^20^^,^^23^ Focusing on their maturation profile within the CNS, we identified a prominent population of ASCs within distinct post-mortem CNS compartments of MS patients and found a relative enrichment in the CNS compared to peripheral blood, which was most profound in MS white matter lesions. Whether this is an MS-specific characteristic is uncertain, since we did not compare with other CNS inflammatory diseases. For example, viral infections of the CNS might involve similar mechanisms of B-cell maturation.^47^, ^48^, ^49^ Recently, high plasmablast frequencies were already detected in MS CSF versus blood,^50^ but we extend these data with our findings. The increased presence of ASCs in the MS CNS supports the hypothesis that CNS-infiltrated memory B cells in MS mature into antibody-producing plasmablasts and plasma cells upon reactivation.^16^^,^^20^^,^^23^ Clonal overlap has been found between B cells in distinct CNS compartments and blood of MS patients, indicating that B cells likely migrate towards the CNS and traffic across the tissue barrier within the CNS.^17^^,^^22^^,^^51^^,^^52^ However, not much is known about the effector functions and maturation of these B-cell populations in the CNS and therefore we closely looked into this.

Intrathecal antibody production in MS is prevalent at diagnosis,^53^ and is likely biologically relevant. The pathology of not only advanced but also most of early MS biopsies and autopsies are characterized by IgG and complement deposition, identified as pattern II lesions.^54^^,^^55^ Supporting the participation of antibodies, MS patients with a pattern II pathology show favourable clinical outcomes upon therapeutic plasma exchange.^56^^,^^57^ Furthermore, antibodies in pattern II patients were found to have an increased reactivity to Nogo-A peptides, which are present on neurons and oligodendrocytes.^58^ Interestingly, complement-dependent demyelinating IgG antibodies were only observed in MS patients and not in healthy controls or patients with other neurological diseases.^59^ Therefore, antibody secretion may have an important role in MS pathology. Consistent with the positive link between ASC presence and lesion activity, Ig gene expression, most importantly IgG, was elevated in active MS lesions. ASC presence within the CNS even correlated with OCB profiles and IgG index, indicating that ASCs are likely responsible for intrathecal IgG production. These observations are supported by previous research, showing that OCBs are resulting from CNS-infiltrated plasmablasts and plasma cells.^50^^,^^60^, ^61^, ^62^ Recent studies revealed that MS patients with limited presence of CD20^+^ B cells and CD138^+^ plasma cells in white matter tissue show reduced intrathecal IgG production, based on IgG CSF/plasma ratios and OCB profiles, and have less severe clinical outcomes.^18^^,^^63^ Therefore, intrathecally produced IgG antibodies are likely associated with an unfavourable pathological profile in MS patients. Combined with our data, ASC presence in the MS CNS may thus be linked to more severe disease activity.

We found that CD45 expression was downregulated on ASCs when moving closer to the brain parenchyma and was lowest within MS white matter lesions. This reduction is most likely related to the maturation process of ASCs, since long-lived plasma cells are found to lose CD45 surface expression during their lifespan, indicating increased persistence.^64^, ^65^, ^66^ Pollok et al. demonstrated that long-lived plasma cells are able to persist in the chronically inflamed CNS, which appears to be provided with survival niches for these cells.^67^ Survival niches within the MS brain likely promote the maturation of local ASCs. In MS brain donors, ASCs within the CSF were dominated by CD138^−^ plasmablasts, although CD138^+^ plasma cells were increased in CSF versus blood samples. Supporting these data, short-lived plasmablasts were previously found to be highly present in MS CSF and were identified as a major effector population that contributes to active MS inflammation.^68^ Furthermore, ASCs within the CSF of MS patients highly expressed CXCR3 compared to peripheral blood, which is consistent with our previous findings showing preferential migration of CXCR3^+^ B cells into the CNS.^23^ The ASCs are thus likely derived from CXCR3^+^ B cells, which is further supported by other studies.^36^^,^^42^^,^^69^ CXCR3 expression on ASCs in MS CSF positively correlated with the IgG index. Accordingly, CXCR3 expression is the highest on IgG(1)^+^ memory B cells and CXCR3 levels on *in vitro*-induced ASCs positively correlate with IgG secretion.^23^^,^^42^ This suggests that CXCR3^+^ ASCs are primarily responsible for intrathecal IgG production in MS patients. This mirrors the increased capacity of CXCR3-expressing IgG^+^ B cells that accumulate in the blood of natalizumab-treated MS patients to develop into ASCs *in vitro*.^23^^,^^36^^,^^42^

We investigated multiple factors that may determine B-cell maturation within the MS CNS, including age. Brioschi et al. demonstrated that so-called peripheral age-associated B cells infiltrate the CNS of aged mice and likely mature into autoantibody-secreting plasma cells.^70^ Fransen et al. showed limited CD20^+^ B-cell presence in the human MS brain with increasing age, which could imply more development into ASCs.^18^ We found relative frequencies of *ex vivo* ASCs to decrease with age in CSF, meninges and white matter of patients with end-stage MS. Interestingly, *ex vivo* ASCs in MS white matter lesions did not correlate with age. MS white matter lesions probably contain a different microenvironment compared to normal(-appearing) white matter, in which other aspects such as T-cell presence are critical for maturation into ASCs.

In the periphery, T_FH_ cells are found to be important for triggering autoreactive B cells to develop into CNS-infiltrating CXCR3(T-bet)^+^ B cells under influence of IFN-γ.^20^^,^^23^ Within the CNS, ectopic lymphoid follicles probably represent a critical site for B-cell reactivation, especially for subsets that have been triggered by IFN-γ as shown in mice.^71^^,^^72^ Locally, IL-21 and CD40L signalling from T_FH_ cells might already be sufficient to induce maturation of memory B cells into ASCs.^23^ The positive correlation between total ASC/B-cell ratios and CD4^+^/CD8^+^ memory T cell ratios in MS white mater lesions, in contrast to white matter, suggested that the presence of CD4^+^ memory T cells is indeed important for B-cell maturation into ASCs within MS lesions. Our immunofluorescence data actually gives an indication that local B- and T-cell interaction in MS lesions may involve follicular-like T cells based on CXCR5 expression. Here, CXCR3^+^ B cells could possibly act as potent antigen-presenting cells to receive signals from CD4^+^ T cells that drive their maturation. Strikingly, we demonstrated that local B-cell maturation may depend on the type of T cell that is present in the MS brain.

This work has some limitations. First, we studied tissues of autopsy samples from aged donors with advanced MS. The extrapolation to living MS donors earlier in the disease is uncertain. Nevertheless, since OCB-presence is frequent at diagnosis, and we earlier showed presence of B cells in MS diagnostic biopsy samples,^18^ our findings likely reflect pathological events occurring already at the onset of MS. Second, we did compare ASC versus B-cell ratios between MS and control donors, but did not include other CNS inflammatory diseases. Since a positive correlation has been found with intrathecal IgG production, increased ASC versus B-cell ratios are at least an inflamed CNS-related phenomenon, but specificity for MS needs to be determined in the near future. Third, the current study is mainly focused on the role of antibody secretion in MS, while not taking into account other functions of the B cell that are biologically relevant, such as cytokine secretion. In addition, the use of liberase for proper digestion of the meninges and brain tissue in our isolation protocol affected the expression levels of CD27, CXCR3 and CD138 on ASCs. Therefore, information on the ASC phenotype in these compartments is limited. The data in this study are static and therefore cannot prove any changes over time. Furthermore, different cohorts of MS patients were used in this study. For example, Ig gene expression and total ASC/B-cell ratios have not been measured within brain tissue from the same MS brain donors, so local B-cell maturation could not be linked directly to local Ig production in the tissue. Lastly, we characterized follicular-like T cells in MS lesions as being dominantly CD4^+^ in our flow cytometric analysis, but we did not use CD4 in our immunofluorescence *in situ* staining. Despite these limitations, our study provides an in-depth analysis of B-cell maturation within paired blood, CSF, meninges and brain tissue from a large and very unique cohort of MS brain donors.

Taken together, our findings support the development of CXCR3^+^ B cells towards lesional ASCs, which is likely mediated by brain-resident CD4^+^ memory T cells, as a critical pathophysiological hallmark for diagnosis of MS. These cells potentially contribute to MS disease progression through both intrathecal and local IgG production. Unlike studies suggesting that oligoclonal IgG responses are related to B and plasma cell clones, we here demonstrate that the CD27^high^CD38^high^ ASC phenotype in CNS compartments of people with MS is linked to *in vivo* IgG production in brain tissues and CSF of these donors. Locally produced antibodies may promote MS pathology via indirect mechanisms, such as formation of immune complexes.^73^^,^^74^ Moreover, these antibodies recognize a wide variety of CNS-derived antigens, making it at least a challenge to study their pathogenicity.^75^ Hence, irrespectively of the antigen recognized, local B-cell maturation into ASCs is a promising and generic target mechanism for future MS therapies, including next-generation inhibitor drugs that are capable of penetrating the brain.^76^^,^^77^

## Contributors

L.B., J.v.L., J.S., and M.M.v.L. contributed to the study concept. L.B., H.J.E., M.J., P.P.A.U., M.J.M., A.F.W.W., C.C.H., M.R.J.M., J.H., and J.v.L acquired and analysed data. L.B. drafted the figures. L.B., J.S., and M.M.v.L. verified and interpreted the underlying data and wrote the manuscript. J.S. and M.M.v.L. designed the research, obtained funding, discussed results and supervised the project team. All authors reviewed and approved the manuscript for final publication.

## Data sharing statement

Data supporting our findings will be made available from the corresponding author on reasonable request.

## Declaration of interests

M.M.v.L. received research support from EMD Serono, Merck, GSK and Idorsia Pharmaceutical Ltd. J.S. received lecture and/or consultancy fees from Biogen, Merck, Novartis and Sanofi-Genzyme. The remaining authors declare no competing interests.
