# Supplementary material for: Selective emergence of antibody-secreting cells in the multiple sclerosis brain
Source: eBioMedicine. 2023 Feb 14;89:104465. doi: 10.1016/j.ebiom.2023.104465 (PMC9958261; doi:10.1016/j.ebiom.2023.104465)
Supplement: Supplementary Material [file mmc1.pdf]

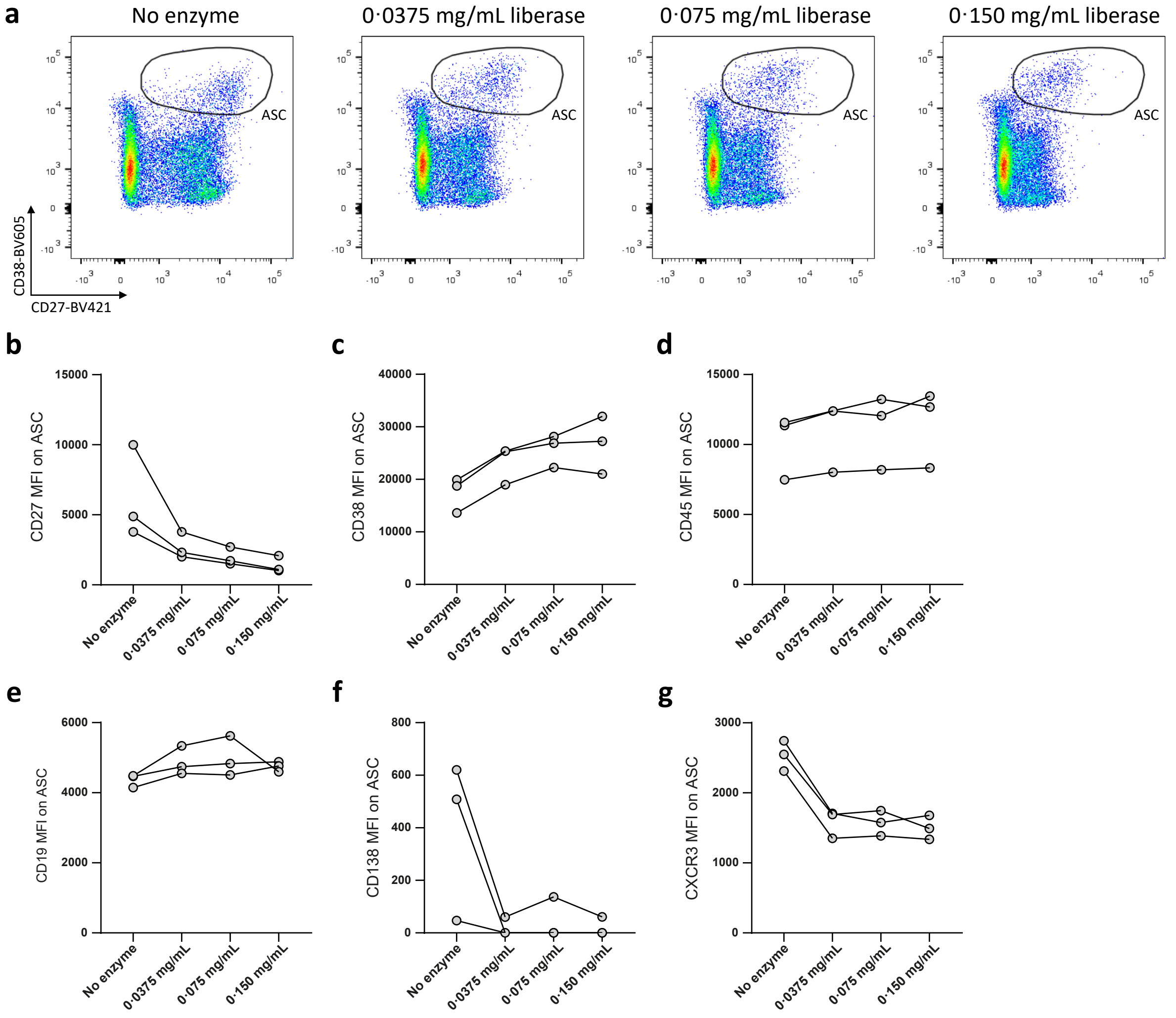

Supplementary Fig. S1: Liberase impacts the expression profiles of CD27, CD138 and CXCR3 on ASCs. PBMCs were freshly isolated from healthy controls (n=3) and treated with [1] 0·0375 mg/mL liberase and 50 U/mL DNase, [2] 0·075 mg/mL liberase and 100 U/mL DNase or [3] 0·150 mg/mL liberase and 200 U/mL DNase. (**a**) Representative flow cytometry dot plots with gating of CD38highCD27high ASCs within viable CD45+CD19+CD3- B cells for all conditions. (**b-g**) Paired MFI's of CD27, CD38, CD45, CD19, CD138 and CXCR3 expression on ASCs for all different conditions. ASC = antibody-secreting cell; MFI = median fluorescence intensity.

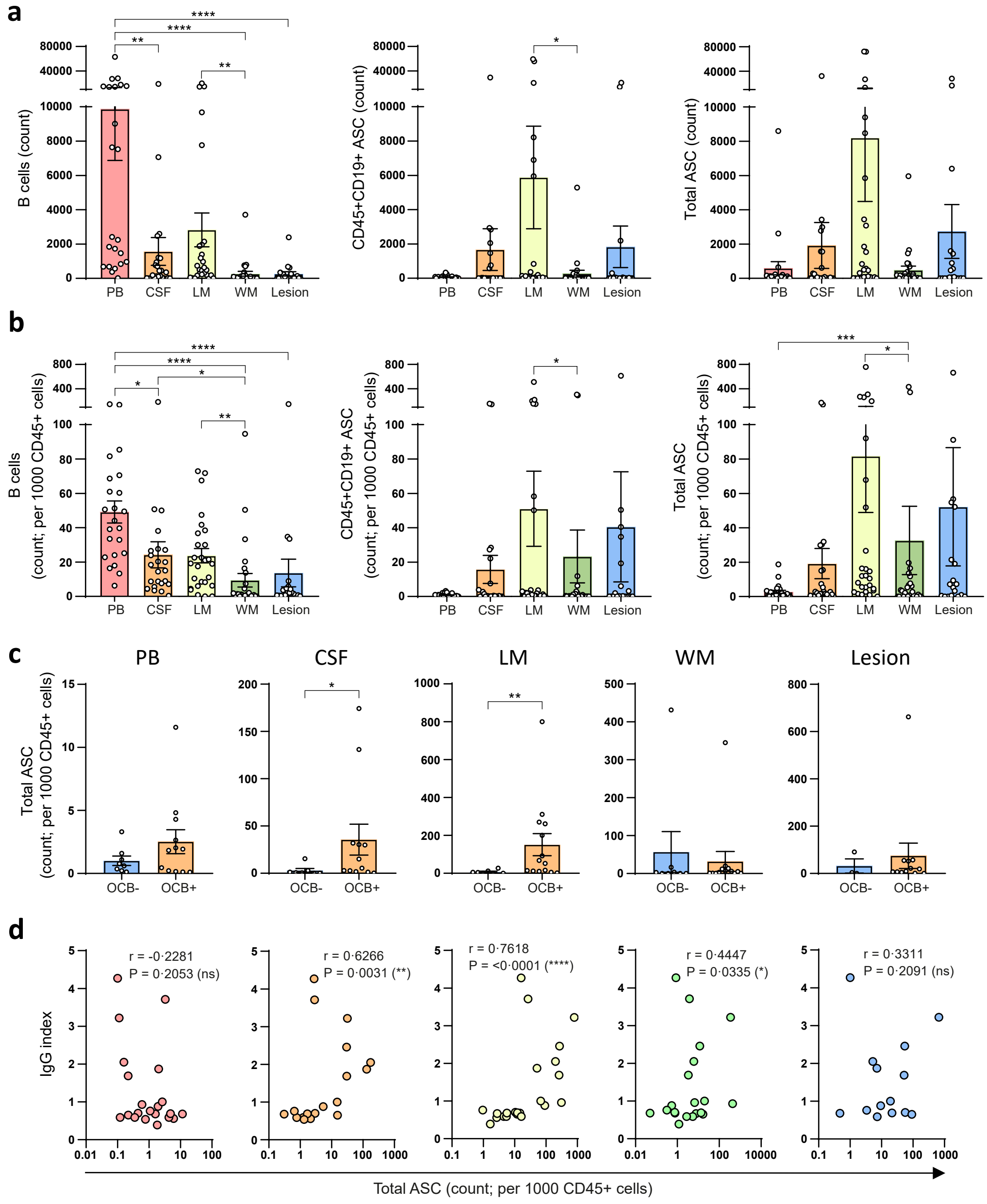

Supplementary Fig. S2: ASC counts are increased in MS CNS compartments and correlate with intrathecal IgG production. (**a-b**) B-cell, CD45+CD19+ ASC and total ASC counts in peripheral and CNS compartments of MS brain donors (n=28), both absolute and corrected for the number of CD45+ lymphocytes. (**c**) Total ASC counts in post-mortem peripheral blood, CSF, leptomeninges, white matter and white matter lesions from OCB-negative and -positive MS patients. (**d**) Correlation plots showing the link between IgG index and total ASC counts in post-mortem peripheral and CNS compartments from MS brain donors. Data are presented as the mean ± standard error of the mean (SEM). p-values were calculated using Kruskal-Wallis and Dunn's post-hoc tests (**a-b**) and Mann-Whitney U tests (**c**). Correlation plots were analysed by calculating Spearman correlation coefficients (**d**). *p<0·05; **p<0·01; ***p<0·001; ****p<0·0001. ASC = antibody-secreting cell; CSF = cerebrospinal fluid; LM = leptomeninges; MS = multiple sclerosis; OCB = oligoclonal band; PB = peripheral blood; WM = white matter.

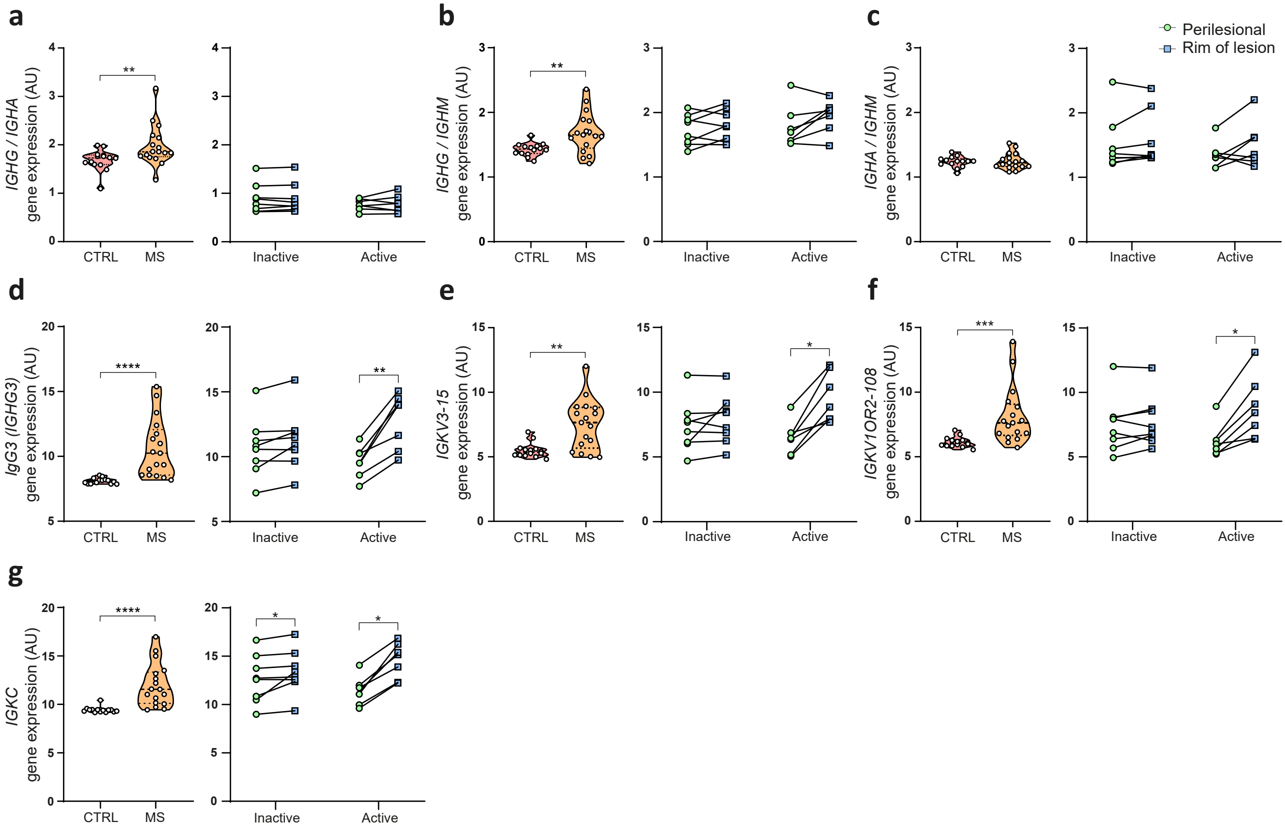

Supplementary Fig. S3: The increase of IgG gene expression in MS brain tissue is dominant over IgM and IgA and is mainly due to IgG1. (**a-c**) Ratios of IGHG / IGHA, IGHG / IGHM and IGHA / IGHM normalized gene expression in post-mortem control white matter (n=14) and MS NAWM (n=17) as well as in post-mortem perilesional tissue and rims of inactive (n=8) and active (n=7) MS white matter lesions, measured with a microarray. (**d-g**) Normalized gene expression levels of IGHG3, IGKV3-15, IGKV1OR2-108 and IGKC in the same brain tissues. Data are presented as the mean ± standard error of the mean (SEM). p-values were obtained by performing Mann-Whitney U tests and Wilcoxon signed-rank tests. *p<0·05; **p<0·01; ***p<0·001; ****p<0·0001. AU = arbitrary units; CTRL = control; MS = multiple sclerosis.

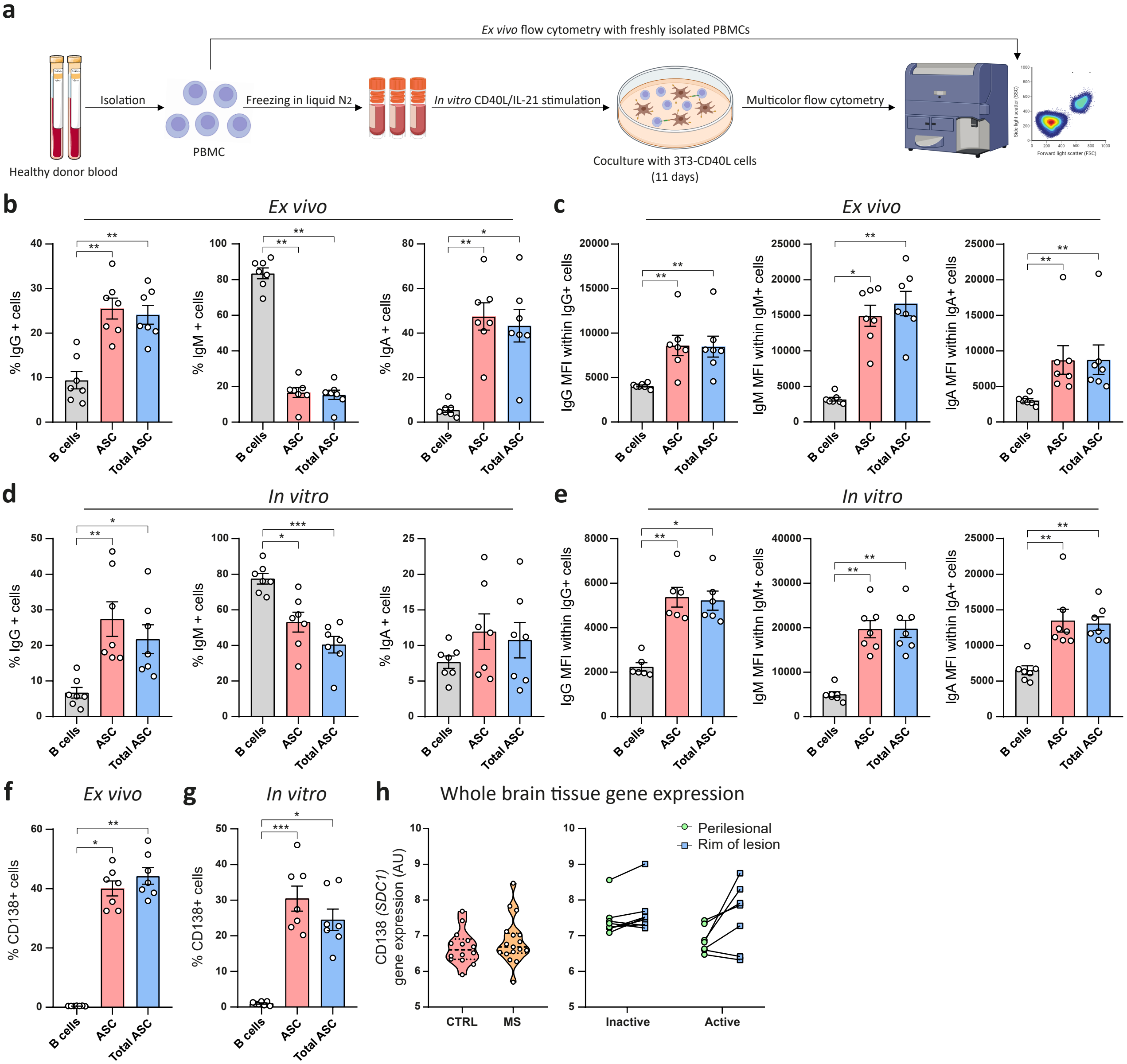

Supplementary Fig. S4: ASCs are high producers of Igs compared to other B cells. (**a**) Schematic overview of the experimental set-up. PBMCs were freshly isolated from peripheral blood of healthy controls (n=7). These ex vivo PBMCs were directly used for multicolour flow cytometry or stored at -80 °C. Subsequently, PBMCs were thawed and cocultured with CD40L-attached 3T3 fibroblasts and soluble IL-21 in vitro. After 11 days of incubation at 37°C and 5% CO_2, the cells were used for flow cytometry. (**b, d**) Percentages of intracellular producing IgG+, IgM+ and IgA+ cells within ex vivo and in vitro B cells (CD45+CD19+CD3-CD38-dim/-), ASCs (CD45+CD19+CD3-CD38highCD27high) and total ASCs (CD38highCD27highCD3-). (**c, e**) Quantifications of intracellular IgG, IgM and IgA MFI's within ex vivo and in vitro IgG+, IgM+ and IgA+ B cells, ASCs and total ASCs. (**f, g**) Percentages of CD138+ cells within ex vivo and in vitro B cells, ASCs and total ASCs. (**h**) Normalized gene expression levels of SDC1 in post-mortem control (n=14) and MS (n=17) NAWM as well as in post-mortem perilesional tissue and rims of inactive (n=8) and active (n=7) MS white matter lesions, measured with a microarray. Data are presented as the mean ± standard error of the mean (SEM). Data were analysed using Kruskal-Wallis and Dunn's post-hoc tests (**b-g**), Mann-Whitney U tests and Wilcoxon signed-rank tests (**h**). *p<0·05; **p<0·01; ***p<0·001; ****p<0·0001. AU = arbitrary units; ASC = antibody-secreting cells; CTRL = control; MFI = median fluorescence intensity; MS = multiple sclerosis; PBMC = peripheral blood mononuclear cell.

Supplementary Table 1: Clinical information of each individual brain donor included for *ex vivo* phenotyping of B cells

Donor number	Clinical diagnosis	Gender	Age in years	Disease duration in years	Therapies during disease course[1]	Cause of death	PMD in hours	pH-value of CSF
D1	MS	Female	82	60	-	Legal euthanasia	8.6	6.46
D2	SPMS	Female	68	35	Prednisolone	Encephalitis, pneumonia	9.1	6.55
D3	SPMS	Male	60	17	Interferon β-1α	Physician-assisted suicide	8.8	6.67
D4	PPMS	Female	66	23	-	Pneumonia	9.8	6.30
D5	PPMS	Female	52	27	Methylprednisolone	Legal euthanasia	8.7	6.64
D6	SPMS	Female	62	31	-	Legal euthanasia	8.7	6.91
D7	PPMS	Female	49	25	Methylprednisolone	Legal euthanasia	8.5	6.34
D8	RRMS	Female	39	8	Methylprednisolone, Glatiramer	MS, coma	8.5	6.26
D9	MS	Female	65	37	Methylprednisolone	Pneumonia	10.3	6.56
D10	SPMS	Male	65	36	Interferon β, Methylprednisolone	Legal euthanasia	9.5	6.50
D11	SPMS	Female	51	17	Glatiramer, Interferon β-1α	Legal euthanasia	9.2	6.87
D12	MS	Female	67	16	-	Legal euthanasia	5.8	6.62
D13	SPMS	Female	50	18	Interferon β-1α, Methylprednisolone	Legal euthanasia	10.3	6.44
D14	PPMS	Female	52	28	-	Legal euthanasia	9.5	6.90
D15	PPMS	Male	56	20	Methylprednisolone, Interferon β	Pneumonia	6.3	6.60
D16	SPMS	Male	71	43	-	Pneumonia	7.3	6.60
D17	PPMS	Male	65	17	Prednisolone	Terminal liver cirrhosis	7.3	6.10
D18	SPMS	Female	68	18	-	Urosepsis	9.3	-
D19	PPMS	Female	53	17	-	Legal euthanasia	5.8	6.76
D20	SPMS	Female	61	22	-	Urosepsis, hydronephrosis	8.1	6.41
D21	SPMS	Male	70	40	-	Dehydration, MS, heart failure	5.2	6.82
D22	SPMS	Female	67	39	Methylprednisolone	Sepsis from infected bile ducts	6.7	6.55
D23	SPMS	Female	65	18	-	MS, metastatic breast cancer	5.6	6.44
D24	SPMS	Female	51	16	Methylprednisolone	Legal euthanasia	8.0	6.40
D25	SPMS	Female	65	33	-	Septic shock, multi-organ failure	8.3	6.25
D26	MS	Female	65	2	-	Legal euthanasia	7.2	6.63
D27	PPMS	Female	82	33	-	Cardiac arrest	7.5	6.30
D28	MS	Female	70	23	Interferon β-1α, Methylprednisolone	Urinary tract infection	6.6	6.40
NDC 1	NO (Polio, PMR)	Female	72	-	-	Legal euthanasia	6.8	7.22
NDC 2	Epi (Braak 1A)	Female	86	-	-	Renal insufficiency	8.1	7.07
NDC 3	PSP (Braak 1)	Female	82	-	-	Unknown	5.5	6.36
NDC 4	PD (Braak 1O)	Male	84	-	-	Cachexia, dehydration	8.3	6.36
NDC 5	FXS (Braak 2A)	Female	84	-	-	Cachexia	8.9	6.74
NDC 6	NO (Heart failure; Braak 1A)	Female	75	-	-	Legal euthanasia	9.2	6.57
NDC 7	PD (Braak 1)	Female	66	-	-	Legal euthanasia	7.2	6.65
NDC 8	NO (Cardiac cancer; Braak 1A)	Male	68	-	-	Cancer	5.8	6.50
NDC 9	NO (Multiple CVA; Braak 2)	Female	88	-	-	Legal euthanasia	6.3	6.52
NDC 10	PD (Braak 3)	Male	92	-	-	Terminal physical deterioration, pneumonia, dehydration	8.8	6.89

[1]Medications have not been administered in the last three months and rarely in the last year before death

CSF = cerebrospinal fluid; CVA = cerebral vascular accident; Epi = epilepsy; FXS = fragile X syndrome; MS = multiple sclerosis; NDC = non-demented control; NO = no brain-related disease; PD = Parkinson’s disease; PMD = post-mortem delay; PMR = polymyalgia rheumatica; PPMS = primary progressive multiple sclerosis; PSP = progressive supranuclear palsy; RRMS = relapsing-remitting multiple sclerosis; SPMS = secondary progressive multiple sclerosis

Supplementary Table 2: Clinical information of each individual brain donor included for transcript analysis on white matter

Donor number	Clinical diagnosis	Gender	Age in years	Disease duration in years	Therapies during disease course[1]	Cause of death	PMD in hours	pH-value of CSF
D1	SPMS	Female	69	25	Methylprednisolone	Respiratory insufficiency	9.3	6.40
D2	SPMS	Female	40	14	Methylprednisolone	Dehydration	7.0	6.74
D3	SPMS	Female	53	18	Prednisone	Pneumonia	7.3	6.54
D4	PPMS	Female	62	25	Prednisone, Interferon	Cardiac asthma	6.8	6.49
D5	SPMS	Female	40	11	Methylprednisolone, Prednisone	Pneumonia	7.0	6.33
D6	SPMS	Female	70	32	-	Cardiac arrest	6.5	6.30
D7	SPMS	Female	76	54	Prednisolone	Respiratory insufficiency	14.3	5.93
D8	SPMS	Female	64	35	Prednisone	Pneumonia, dehydration	7.8	6.22
D9	SPMS	Female	58	20	Methylprednisolone	Legal euthanasia	8.2	6.30
D10	SPMS	Female	71	23	Methylprednisolone, Prednisone	Pneumonia	8.0	6.80
D11	SPMS	Female	71	31	Prednisone	Respiratory insufficiency	10.4	6.35
D12	RRMS	Female	38	10	Methylprednisolone	Cardiac arrest	5.3	6.55
D13	SPMS	Female	69	27	-	Viral infection	13.3	6.12
D14	SPMS	Female	48	9	-	Legal euthanasia	8.2	6.55
D15	SPMS	Female	66	43	Prednisone, Methylprednisolone	Liver failure	6.3	6.44
D16	SPMS	Female	80	58	Methylprednisolone	Acute leukemia	9.6	6.20
D17	SPMS	Female	48	22	Prednisone, Methylprednisolone	Heart failure	5.8	6.64
NDC 1	NO (CVA; Braak 2B)	Female	74			Cachexia	6.7	6.70
NDC 2	NO (Braak 0)	Female	54			Acute renal failure	8.0	6.45
NDC 3	NO (Metastasized NSCLC; Braak 2)	Female	71			Cardiac arrest	4.8	6.70
NDC 4	NO (Braak 2C)	Female	60			Cardiac arrest	8.4	6.6
NDC 5	NO (CML; Braak 2)	Female	69			Respiratory insufficiency	7.2	9.80
NDC 6	NO (NP; Braak 1)	Female	65			Cardiac arrest	12.8	6.90
NDC 7	NO (Metastasized ovarium cancer; Braak 1)	Female	61			Pneumonia, cachexia	7.3	7.20
NDC 8	NO (Lung bleeding caused by tumor; Braak 0)	Female	68			Legal euthanasia	5.8	6.97
NDC 9	NO (LMS; Braak 0)	Female	52			Legal euthanasia	6.5	7.20
NDC 10	NO (Metastasized vaginal cancer; Braak 1A)	Female	41			Pulmonary artery hemorrhage	13.5	-
NDC 11	NO (Heart failure; Braak 0)	Male	56			Unknown	14.0	7.03
NDC 12	NO (Braak 0)	Male	56			Myocardial infarction	9.3	6.54
NDC 13	NO (Metastasized lung cancer; Braak 1O)	Female	50			Unknown	4.2	6.98
NDC 14	NO (Metastasized pancreas cancer; Braak 1O)	Male	62			Unknown	7.3	6.36

[1]Medications have not been administered in the last three months and rarely in the last year before death
CML = chronic myelogenous leukemia; CSF = cerebrospinal fluid; CVA = cerebral vascular accident; LMS = leiomyosarcoma; NO = no brain-related disease; NP = necrotic pneumonia; NSCLC = non-small cell lung cancer; PMD = post-mortem delay; PPMS = primary progressive multiple sclerosis; RRMS = relapsing-remitting multiple sclerosis; SPMS = secondary progressive multiple sclerosis

Supplementary Table 3: Clinical information of each individual brain donor included for transcript analysis on MS lesions

Donor number	Clinical diagnosis	Gender	Age in years	Lesion type	Disease duration in years	Therapies during disease course[1]	Cause of death	PMD in hours	pH-value of CSF
D1	SPMS	Female	66	I	43	Prednisone, Methylprednisolone	Liver failure	6.3	6.44
D2	SPMS	Female	75	I	42	Methylprednisolone	Pneumonia	8.0	6.50
D3	PPMS	Female	72	I	14	Prednisone	Pneumonia	12.0	6.85
D4	SPMS	Female	64	I	39	Methylprednisolone	Urosepsis	10.2	6,30
D5	PPMS	Female	77	I	29	Prednisolone, Methylprednisolone	Legal euthanasia	10.0	6.50
D6	PPMS	Male	45	I	19	Methylprednisolone, Interferon β-1α	Pulmonary embolism	7.8	6.22
D7	PPMS	Male	50	I	24	Methylprednisolone, Interferon β-1α	Unknown	9.5	6.20
D8	MS	Female	57	I	29	-	Legal euthanasia	8.7	6.44
D9	SPMS	Female	53	CA	18	Prednisone	Pneumonia	7.3	6.54
D10	SPMS	Female	40	CA	11	Methylprednisolone, Prednisone	Pneumonia	7.0	6.33
D11	SPMS	Female	45	CA	14	Methylprednisolone	Legal euthanasia	10.9	6.62
D12	SPMS	Female	48	CA	9	-	Legal euthanasia	8.2	6.55
D13	PPMS	Male	43	CA	17	Methylprednisolone	Pneumonia	8.7	6.48
D14	SPMS	Female	66	CA	23	-	Unknown	6.0	6.18
D15	SPMS	Male	51	CA	20	Methylprednisolone, Interferon β-1α	Unknown	11.0	6.23

[1]Medications have not been administered in the last three months and rarely in the last year before death
CA = chronic active; CSF = cerebrospinal fluid; I = inactive; MS = multiple sclerosis; SPMS = secondary progressive multiple sclerosis; PMD = post-mortem delay; PPMS = primary progressive multiple sclerosis

Supplementary Table 4: Clinical information of each individual brain donor included for immunofluorescence staining

Donor number	Clinical diagnosis	Gender	Age in years	Disease duration in years	Therapies during disease course[1]	Cause of death	PMD in hours	pH-value of CSF
D1	MS	Female	53	23	Methylprednisolone, Interferon β	Euthanasia	10.8	6.86
D2	MS	Female	66	23	-	Unknown	6.0	6.18
D3	MS	Male	53	27	-	Pneumonia, cachexia, dehydration	10.0	6.38
D4	MS	Male	56	33	Prednisolone	End stage MS with urosepsis	9.6	6.44
D5	MS	Female	53	17	Methylprednisolone	Euthanasia	7.3	6.81
D6	MS	Male	48	16		Ileus and dehydration	6.6	6.29

[1]Medications have not been administered in the last three months and rarely in the last year before death
CSF = cerebrospinal fluid; MS = multiple sclerosis; PMD = post-mortem delay

Supplementary Table 5: Clinical information of each individual brain donor included for *ex vivo* phenotyping of T cells

Donor number	Clinical diagnosis	Gender	Age in years	Disease duration in years	Therapies during disease course[1]	Cause of death	PMD in hours	pH-value of CSF
D1	MS	Female	73	51	Methylprednisolone	Euthanasia	7.8	6.50
D2	MS	Female	60	23	Methylprednisolone, Interferon β	Euthanasia	5.1	6.80
D3	MS	Male	71	49	Prednisone	Respiratory insufficiency	6.9	6.40
D4	MS	Male	51	18	Fingolimod, Glatiramer, Interferon β, prednisone	Dehydration	6.1	6.55

NBB no. = brain donor number that is assigned by the Netherlands Brain Bank
PMD = post mortem delay; CSF = cerebrospinal fluid; MS = multiple sclerosis

Supplementary Table 6: Fluorochrome-labelled monoclonal anti-human antibodies used for flow cytometry

Antibody marker	Fluorochrome	Clone	RRID	Catalogue number	Company
CD3	FITC	SK7	-	345763	BD Biosciences[1]
CD3	AF700	SK7	AB_2563420	344822	Biolegend[2]
CD3	APC-H7	SK7	-	641415	BD Biosciences
CD3	BV786	SK7	AB_2744384	563800	BD Biosciences
CD3	PE-CF594	UCHT1	-	15821809	eBioscience[3]
CD4	BV510	OKT4	AB_2561866	317444	Biolegend
CD4	BUV563	SK3	AB_2739451	612912	BD Biosciences
CD8	FITC	SK1	-	345772	BD Biosciences
CD8	PerCP-Cy5.5	SK1	-	341050	BD Biosciences
CD8	BV786	RPA-T8	AB_2687487	563823	BD Biosciences
CD19	BV786	HIB19	AB_2563442	302240	Biolegend
CD27	BV421	M-T271	AB_11153497	562513	BD Biosciences
CD38	PE	HB7	AB_2561900	356604	Biolegend
CD38	PE-Cy7	HIT2	AB_2072782	303516	Biolegend
CD38	BV605	HIT2	AB_2562915	303532	Biolegend
CD38	PerCP-Cy5.5	HIT2	AB_893314	303522	Biolegend
CD45	PerCP-Cy5.5	HI30	AB_893338	304028	Biolegend
CD45	FITC	HI30	AB_395874	555482	BD Biosciences
CD45RA	APC-H7	HI100	AB_1727497	560674	BD Biosciences
CD45RA	BV711	HI100	AB_2738392	563733	BD Biosciences
CD69	BV650	FN50	AB_2563158	310934	Biolegend
CD103	BUV395	Ber-ACT8	AB_2738759	564346	BD Biosciences
CD138	PE-CF594	MI15	AB_2738863	564606	BD Biosciences
CD138	APC	MI15	AB_2744283	566050	BD Biosciences
CD138	BV605	MI15	AB_2738122	563294	BD Biosciences
CCR7	PE-CF594	150503	AB_11153301	562381	BD Biosciences
CXCR3	APC	G025H7	AB_10983064	353708	Biolegend
CXCR3	PE-Cy7	G025H7	AB_11219383	353720	Biolegend
CXCR3	PE-CF594	G025H7	AB_2564288	353736	Biolegend
CXCR5	AF488	RF8B2	AB_397034	558112	BD Biosciences
IgM	BV510	MHM-88	AB_2562916	314522	Biolegend
IgG	APC-H7	G18-145	AB_10611877	561297	BD Biosciences
IgA	FITC	IS11-8E10	-	130-114-001	Miltenyi Biotech[4]
Fixable viability dye	eFluor520	-	-	65-0867-18	eBioscience
Fixable viability dye	AF700	-	-	564997	BD Biosciences
Fixable viability dye	eFluor780	-	-	65-0865-14	Thermo Fisher Scientific

[1] BD Biosciences, Erembodegem, Belgium [2] Biolegend, London, United Kingdom [3] eBioscience, San Diego, United States [4] Miltenyi Biotech, Leiden, the Netherlands [5] Thermo Fisher Scientific, Waltham, United States

Supplementary Table 7: Criteria for distinguishing different stages of MS white matter

Stage	Perivascular infiltrates	Myelin	HLA-DP/DQ/DR
**0. NAWM**	-	+	-
**1. Reactive site**	±	+	+
**2. Active lesion**	±	±	+ (throughout lesion)
**3. Mixed active/inactive lesion**	±	±	+ (at lesion border)
**4. Inactive lesion**	±	-	-

HLA-DP/DQ/DR = human leukocyte antigen DP/DQ/DR
